# A Four-MicroRNA Panel in Serum as a Potential Biomarker for Screening Renal Cell Carcinoma

**DOI:** 10.3389/fgene.2022.897827

**Published:** 2022-07-22

**Authors:** Rongkang Li, Chong Lu, Xinji Li, Xuan Chen, Guocheng Huang, Zhenyu Wen, Hang Li, Lingzhi Tao, Yimin Hu, Zhengping Zhao, Zebo Chen, Yongqing Lai

**Affiliations:** ^1^ Department of Urology, Guangdong and Shenzhen Key Laboratory of Male Reproductive Medicine and Genetics, Peking University Shenzhen Hospital, Clinical College of Anhui Medical University, Shenzhen, China; ^2^ The Fifth Clinical Medical College of Anhui Medical University, Hefei, China; ^3^ Shantou University Medical College, Shantou, China

**Keywords:** microRNA, renal cell carcinoma, biomarker, bioinformatics, diagnosis

## Abstract

**Background:** Renal cell carcinoma (RCC) has been a major health problem and is one of the most malignant tumors around the world. Serum microRNA (miRNA) profiles previously have been reported as non-invasive biomarkers in cancer screening. The aim of this study was to explore serum miRNAs as potential biomarkers for screening RCC.

**Methods:** A three-phase study was conducted to explore serum miRNAs as potential biomarkers for screening RCC. In the screening phase, 12 candidate miRNAs related to RCC were selected for further study by the ENCORI database with 517 RCC patients and 71 NCs. A total of 220 participants [108 RCC patients and 112 normal controls (NCs)] were enrolled for training and validation. The dysregulated candidate miRNAs were further confirmed with 30 RCC patients and 30 NCs in the training phase and with 78 RCC patients and 82 NCs in the validation phase. Receiver operating characteristic (ROC) curves and the area under the ROC curve (AUC) were used for assessing the diagnostic value of miRNAs. Bioinformatic analysis and survival analysis were also included in our study.

**Results:** Compared to NCs, six miRNAs (miR-18a-5p, miR-138-5p, miR-141-3p, miR-181b-5p, miR-200a-3p, and miR-363-3p) in serum were significantly dysregulated in RCC patients. A four-miRNA panel was built by combining these candidate miRNAs to improve the diagnostic value with AUC = 0.908. ABCG1 and RNASET2, considered potential target genes of the four-miRNA panel, may play a significant role in the development of RCC.

**Conclusion:** A four-miRNA panel in serum was identified for RCC screening in our study. The four-–miRNA panel has a great potential to be a non-invasive biomarker for RCC screening.

## Introduction

Renal cell carcinoma (RCC) is the seventh most common tumor in developed countries and is a major health problem, accounting for approximately 3% of all cancers (Ferlay et al., 2018; Padala et al., 2020). There were an estimated 431,288 new cases and 179,368 deaths of RCC around the world in 2020 (Sung et al., 2021). Clear-cell renal cell carcinoma (ccRCC) is the most common subtype, accounting for 80–90% of kidney cancers (Padala et al., 2020). Most of the RCC patients remain asymptomatic until they advanced. The typical triad: abdominal pain, visible hematuria, and palpable abdominal masses are rare today. The gold standard for the diagnosis of kidney cancer is pathological biopsy, which is an invasive method. This method is generally not suitable for screening (Ljungberg et al., 2019). Some RCCs are accidentally discovered through abdominal computed tomography (CT), but CT cannot be a routine screening method with its radioactivity (Ma et al., 2017). A large number of molecular markers have been studied, but these techniques have not improved the current prognostic system (Lopez-Beltran et al., 2018). Also, the survival rate of RCC depends on the clinical stage at diagnosis, with a 5-year survival rate of about 90% for localized tumors and 12% for metastatic tumors (Padala et al., 2020). Therefore, we need to identify better disease markers. Such biomolecules will also help improve diagnostic and prognostic capabilities and options for possible targets for new treatments.

The ideal biomarker molecule should meet the following characteristics: the ability to isolate them through a non-invasive and inexpensive process; high specificity for different pathological conditions; demonstrate high diagnostic sensitivity; the biological rationality of underlying disease-related mechanisms; and robustness in order to allow routine clinical applications (Al-Shaheri et al., 2021). More and more shreds of evidence showed that microRNAs (miRNAs) have these characteristics. MicroRNAs are small and non-coding RNA molecules about 22 nucleotide-long and underlie the epigenetic regulation of gene expression (Bartel, 2004; He and Hannon, 2004). Many miRNAs have been found to be related to cancer, either as tumor suppressors or as oncogenes. MiRNAs that target negative regulators of carcinogenic pathways may also cause cancer when they are abnormally regulated (Goodall and Wickramasinghe, 2021). Now, many reports also showed that miRNA expression signatures from tumor tissue or liquid biopsy can make a more accurate diagnosis and prognosis for cancer patients (Bracken et al., 2016). In summary, serum miRNA is promising as a biomarker for RCC screening.

To identify potential RCC screening biomarkers, the study was divided into three phases. Through transcription-polymerase chain reaction (qRT-PCR), we evaluated the expression profile of serum miRNA and evaluated the value of key miRNAs in the screening of RCC. By using bioinformatics methods, we also researched the biological functions of critical miRNAs in this study.

## Materials and Methods

### Participants and Ethics Statement

From December 2017 to April 2021, our study recruited 220 participants, including 108 RCC patients and 112 normal controls (NCs) at the Peking University Shenzhen Hospital with the approval of the Ethics Committee of Peking University Shenzhen Hospital. All the participants understood and then signed the informed consent form voluntarily, and the collection process of the serum sample observed the relevant regulations issued by the committee. The patients diagnosed with RCC were based on histology, receiving no treatment before specimen collection. Normal controls are healthy volunteers without a history of cancer and other diseases.

### Research Design

Select RCC-related miRNAs as candidate biomarkers from research studies published on the Gene Expression Omnibus and on the PubMed database. Then, we conducted a three-phase study to determine these candidate biomarkers. First, the expression level of these miRNAs was screened out in the Encyclopedia of RNA Interactomes (ENCORI) database with 517 RCC patients and 71 NCs in the screening phase (Li et al., 2014). These candidate miRNAs were chosen under the standard of *p*-value < 0.01 and fold change (FC) > 2 or <—2 based on the expression level. Then, in the training phase, 30 serum samples from RCC patients and 30 serum samples from NCs were randomly selected to analyze the expression profiles of these candidate miRNAs by using the qRT-PCR method. Finally, in the verification phase, we focused on confirming the expression profiles and diagnostic ability of these candidate miRNAs and constructing the panel with the highest diagnostic ability. Last, bioinformatic analysis and survival analysis were also included in our study. The study flowchart is shown in Supplementary Figure S1.

### Collect Serum Samples and Extract RNA

Before taking serum samples, all participants in this project did not receive any treatment, and 2ul miR-54 (cel-miR-54-5p) (10 nm/L, RiboBio, China) was added to each sample which was used as an internal reference for the RT-qPCR process and normalize the variability in the extraction process. Then, the TRIzol LS isolation kit (Thermo Fisher Scientific, Waltham, MA, United States) was used to extract total RNA from serum samples and NanoDrop 2000c spectrophotometer (Thermo Scientific, United States) was used to measure RNA concentration and purity.

### Quantitative Reverse Transcription-Polymerase Chain Reaction

The expression level of these miRNAs was detected by RT-qPCR. In reverse transcription, the reverse transcription-specific primers of the Bulge-Loop miRNA qRT-PCR primer set (RiboBio, Guangzhou, China) were used to amplify miRNAs. Then, the expression level of miRNAs was observed on the LightCycler480 Real-Time PCR system (Roche Diagnostics, Mannheim, Germany) by the quantitative real-time polymerase chain reaction (qPCR) with the Taqman probe. The qPCR reaction was followed at 95°C for 20 s, 95°C for 10 s, 60°C for 20 s, and 70°C for 10 s, with 40 cycles. Finally, the relative expression level of candidate miRNA was analyzed by the 2-ΔΔCq method (Livak and Schmittgen, 2001).

### Bioinformatic and Prognostic Analysis

MiRWalk3.0 is a database providing the prediction of miRNA target gene interaction with good accuracy (Sticht et al., 2018) and was used for predicting the target genes of candidate miRNAs related to RCC. Only those genes predicted by more than two candidate miRNAs were selected as targeted genes. And then targeted genes were put into the Enrichr database, a gene set search engine that enabled gene set enrichment analysis to conduct the GO functional annotation and KEGG pathway enrichment analysis (Wang et al., 2014; McGeary et al., 2019; Xie et al., 2021). Kaplan–Meier survival analysis and the log-rank test of these candidate miRNAs were conducted by the ENCORI database to explore the overall survival rate of RCC patients (Li et al., 2014).

### Statistical Analysis

The information on demographic and clinical characteristics was expressed as count percentages or mean value ± standard deviation if there were continuous variables between different groups. corresponding tests or analyses were adopted to deal with data. We used the Kruskal–Wallis test to perform multiple comparisons between different independent phases. We used Students’ *t*-test or Mann–Whitney test to analyze the different expression levels of miRNA between RCC patients and NC samples. The miRNA panel was set up by multiple logistic regression analysis. The diagnostic ability of candidate miRNAs was assessed by receiver operating characteristic (ROC) curves and the area under the ROC curve (AUC). The Youden index (calculated as J = Sensitivity + Specificity − 1) was used to determine the optimal sensitivity and specificity. *p*-value less than 0.05 was set as statistical significance (Ge et al., 2021). GraphPad Prism 8 (GraphPad Software Inc., LaJolla, CA), Medcalc (Version 19), and SPSS software (SPSS 26.0 Inc., Chicago, IL) were used to analyze the data.

## Results

### The Clinical and Demographic Characteristics of Participants

After the discovery of candidate miRNAs in the screening phase, a total of 220 participants, including 108 RCC patients and 112 normal controls, were recruited for our study for further training and validation. Fuhrman grade was used for histological classification and the American Joint Committee on Cancer anatomic stage was used for confirming the clinical staging. All normal controls were healthy volunteers without a history of cancer or other diseases. The RCC patients matched normal controls based on age and gender. There was no significant difference in the distribution of age and gender between RCC patients and NCs with *p*-values greater than 0.05. The clinical and demographic characteristics of 220 participants are listed in Table 1.

**TABLE 1:** Demographic and clinical manifestation of 220 participants (RCC and NCs).

	Training phase (*n* = 60)			Validation Phase (*n* = 160)		
	RCC	NC		RCC	NC	
Total number	30	30		78	82	
Age at diagnosis			*p* = 0.64			*p* = 0.24
	48.5 ± 12.2	49.9 ± 11.1		51.6 ± 13.1	54.1 ± 13.8	
Gender			*p* = 0.61			*p* = 0.13
Male	18 (60.0%)	16 (53.3%)		51 (65.4%)	44 (53.7%)	
Female	12 (40.0%)	14 (46.7%)		27 (34.6%)	38 (46.3%)	
Location						
Left	15 (50.0%)			42 (53.8%)		
Right	15 (50.0%)			36 (46.2%)		
Fuhrman Grade						
Grade I	4 (13.3%)			12 (15.4%)		
Grade II	16 (53.3%)			45 (53.8%)		
Grade III	8 (26.7%)			18 (25.0%)		
Grade IV	2 (6.7%)			3 (4.2%)		
AJCC clinical stage						
Stage I	23 (76.7%)			57 (73.1%)		
Stage II	4 (13.3%)			13 (16.7%)		
Stage III	2 (6.7%)			5 (6.4%)		
Stage IV	1 (3.3%)			3 (3.8%)		

During the training phase and validation phase, there was no significant difference between RCCs and NCs in age and gender. Parameters were shown as numbers (percentage). Statistical contrast was exerted through the Wilcoxon–Mann Whitney test.

### Discovery of Candidate miRNAs in the Screening Phase

**FIGURE 1:**
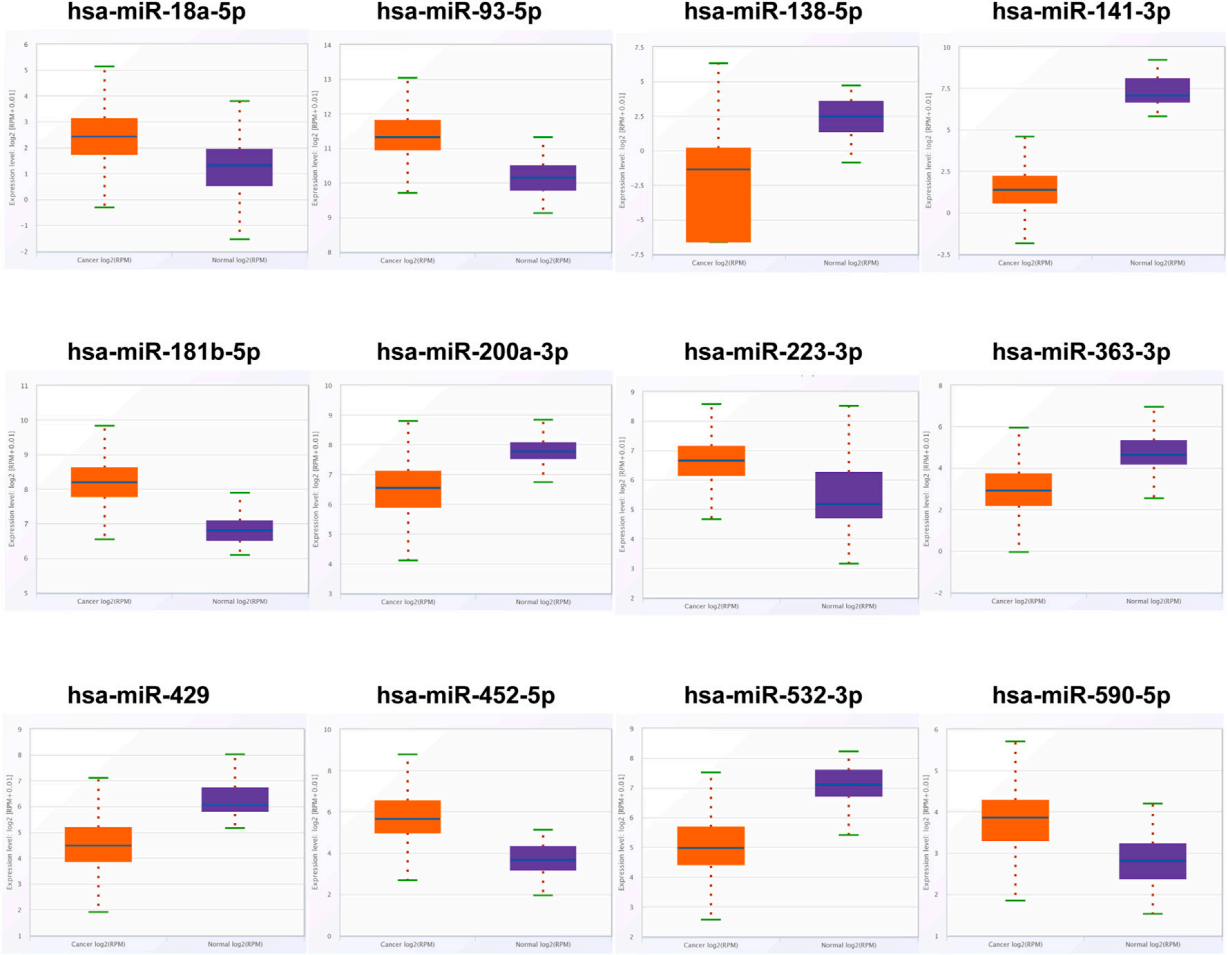
In the training phase, the relative expression levels of the 12 candidate miRNAs. The serums of 30 RCC patients and 30 NCs were adopted in this stage. * represents *p* < 0.05, **represents *p* < 0.01, *** represents *p* < 0.001.

We searched the miRNAs related to RCC from studies on PubMed and then screened out the expression level of these miRNAs in the ENCORI database. Under the standard of *p*-value < 0.01 and fold change (FC) > 2 or <−2 based on the expression level, 12 miRNAs were differentially expressed between RCC patients and NCs (Figure 1; Supplementary Table S2). Thus, 12 miRNAs among which six miRNAs were upregulated significantly and six miRNAs were downregulated significantly were chosen as candidate miRNAs for further study in the next stage.

### Candidate miRNAs in the Training Phase

**FIGURE 2:**
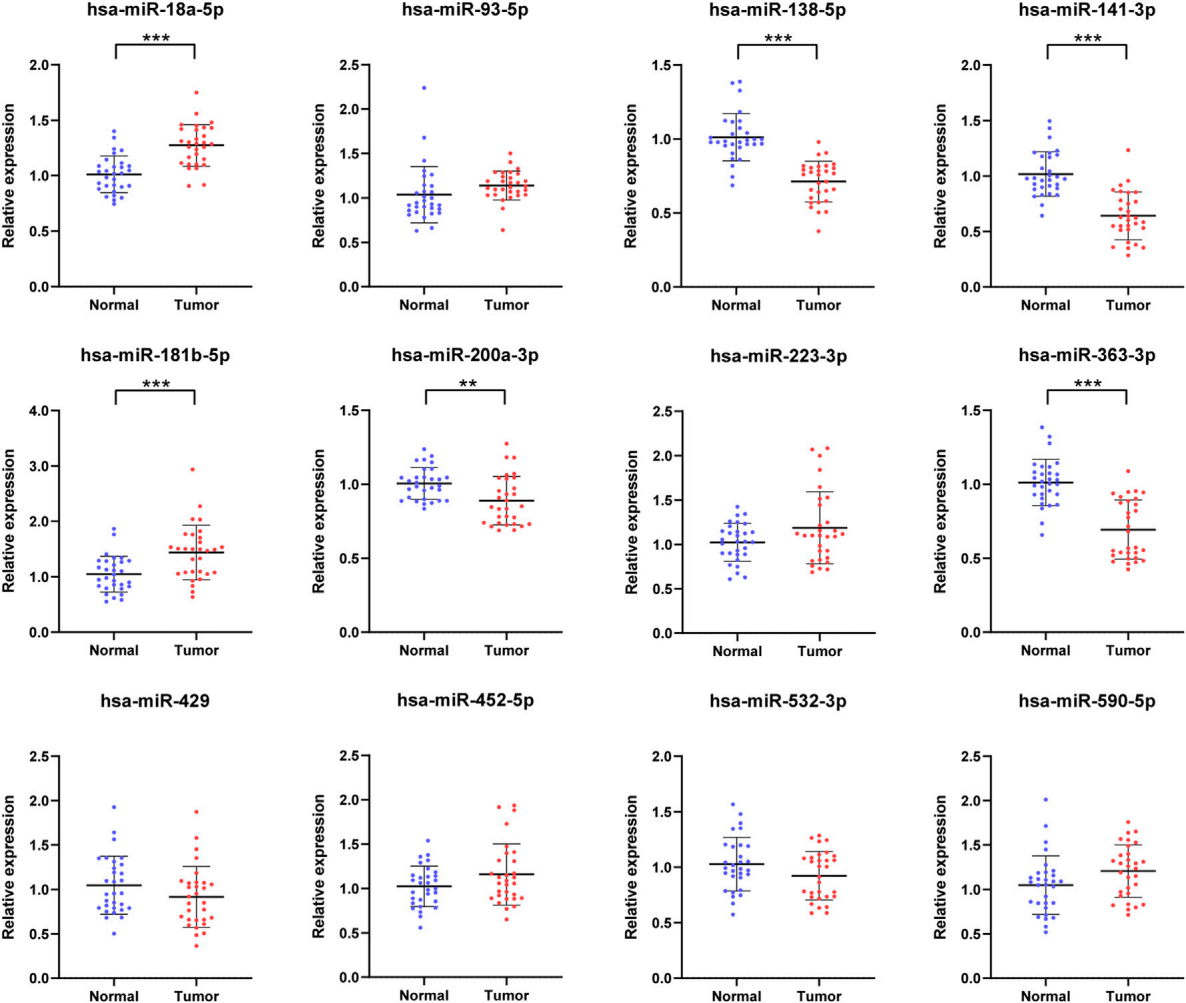
In the training phase, the relative expression levels of the 12 candidate miRNAs. The serums of 30 RCC patients and 30 NCs were adopted in this stage. * represents *p* < 0.05, **represents *p* < 0.01, *** represents *p* < 0.001.

The 12 initial candidate miRNAs were further confirmed with 30 NCs and 30 RCC patients by means of qRT-PCR analysis in the training phase. As shown in Figure 2, the expression level of six miRNAs (miR-18a-5p, miR-138-5p, miR-141-3p, miR-181b-5p, miR-200a-3p and miR-363-3p) in serum, among the 12 candidate miRNAs, were still dysregulated eminently between RCC patients and NCs with *p*-value < 0.05. Also, the pattern of expression in the training phase was the same as in the screening phase.

### Candidate miRNAs and Ability of Diagnosing Renal Cell Carcinoma in the Validation Phase

**FIGURE 3:**
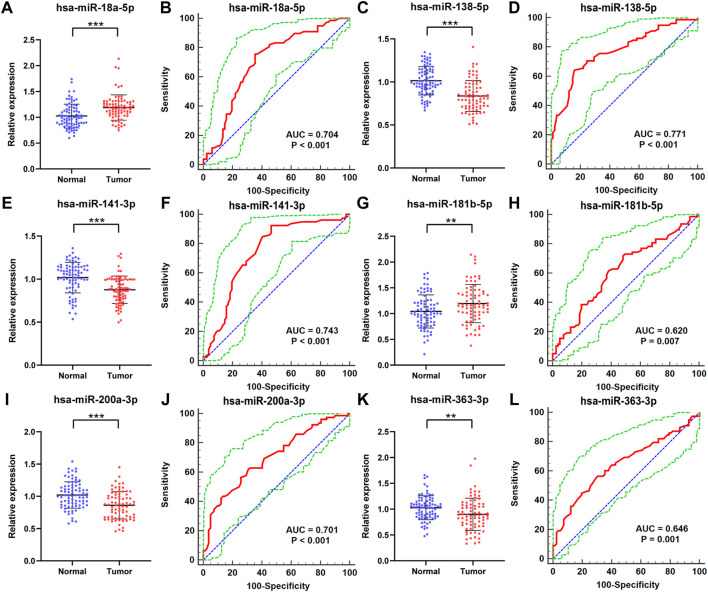
In the validation phase, the relative expression levels and receiver operating characteristic (ROC) curve analyses of six miRNAs with 78 RCC patients and 82 NCs. The relative expression level of **(A)** miR-18a-5p, **(C)** miR-138-5p, **(E)** miR-141-3p, **(G)** miR-181b-5p, **(I)** miR-200a-3p, **(K)** miR-363-3p in serum between normal controls and RCC patients. ROC curve analyses of **(B)** miR-18a-5p, **(D)** miR-138-5p, **(F)** miR-141-3p, **(H)** miR-181b-5p, **(J)** miR-200a-3p, **(L)** miR-363-3p. * represents *p* < 0.05, **represents *p* < 0.01, and *** represents *p* < 0.001. The red curve represents the receiver operating characteristic (ROC) curve. The green curve represents 95% ROC confidence interval. The blue line represents the diagonal.

With additional 78 RCC patients and 82 NCs, we further assessed the six candidate miRNAs to verify the expression of the candidate miRNAs in serum for potential use as biomarkers in RCC screening. As shown in Figure 3, the relative expression level of these candidate miRNAs was still to be dysregulated with *p*-value <0.05. The expression levels of miR-18a-5p and miR-181b-5p were overexpressed in RCC patients compared with the NCs, while the other four miRNAs, including miR-138-5p, miR-141-3p, miR-200a-3p, and miR-363-3p, showed an opposite result, same as in the training phase.

Furthermore, the Receiver Operating Characteristic (ROC) curves of the six candidate miRNAs were performed to assess their respective diagnostic capabilities. The AUCs were listed as follows: the AUCs were 0.704 (95% confidence interval (CI): 0.627 to 0.773; Figure 3B) for miR-18a-5p, 0.771 (95% CI: 0.698 to 0.834; Figure 3D) for miR-138-5p, 0.743 (95% CI: 0.668–0.808; Figure 3F) for miR-141-3p, 0.620 (95% CI: 0.540 to 0.696; Figure 3H) for miR-181b-5p, 0.701 (95% CI: 0.623 to 0.771; Figure 3J) for miR-200a-3p, and 0.646 (95% CI: 0.567 to 0.720; Fig 3L) for miR-363-3p. Moreover, Youden index was performed to calculate optimum cutoff values and the best specificity and sensitivity of these six candidate miRNAs in diagnosing RCC are shown in Table 2.

**TABLE 2:** Outcomes of receiver operating characteristic curves and Youden index for six candidate miRNAs and the panel.

	AUC	*p*-Value	95% CI	Associated criterion	Sensitivity (%)	Specificity (%)
miR-18a-5p	0.704	< 0.001	0.627–0.773	>1.05	75.64	64.63
miR-138-5p	0.771	< 0.001	0.698–0.834	≤0.85	64.10	84.15
miR-141-3p	0.743	< 0.001	0.668–0.808	≤1.03	92.31	53.66
miR-181b-5p	0.620	0.0073	0.540–0.696	>1	73.08	50.62
miR-200a-3p	0.701	< 0.001	0.623–0.771	≤0.91	62.82	68.29
miR-363-3p	0.646	< 0.001	0.567–0.720	≤0.9	56.41	69.51
four-miRNA panel	0.908	< 0.001	0.852–0.948	>0.52483	80.77	88.89

AUC, area under curve; CI, confidence interval.

### Building up miRNA Panels for Better Detection of Renal Cell Carcinoma

**FIGURE 4:**
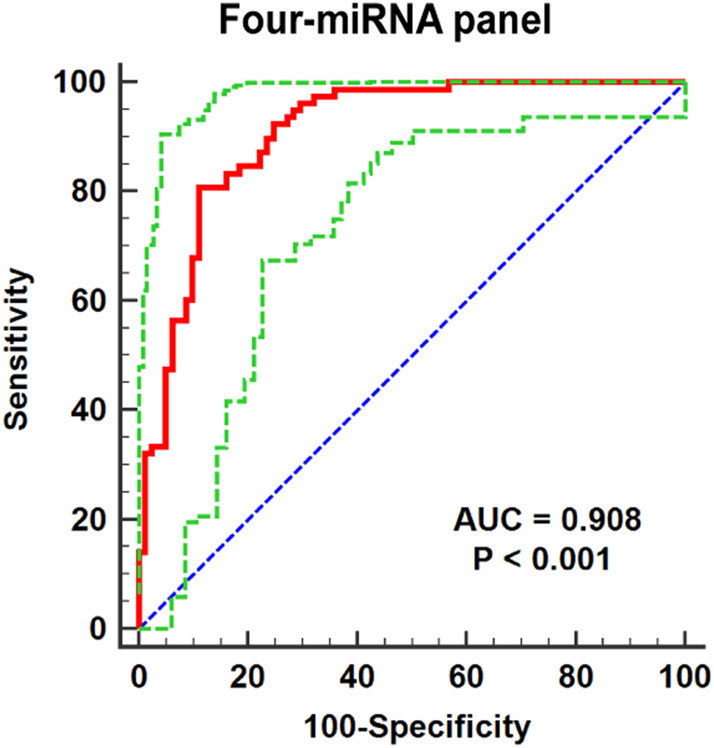
Receiver operating characteristic (ROC) curve analyses for the four-miRNA panel (miR-18a-5p, miR-138-5p miR-141-3p, and miR-181b-5p). The AUC for the panel was 0.908 (95% CI: 0.852–0.948; sensitivity = 80.77%, specificity = 88.89%). The red curve represents the receiver operating characteristic (ROC) curve. The green curve represents 95% ROC confidence interval. The blue line represents diagonal.

One single miRNA might be performed to discriminate RCC patients from NCs, while with a low sensibility and specificity. Thus, it was necessary to combine these representative miRNAs and construct the miRNA panels for enhancing accuracy in the detection of RCC. Through the stepwise logistic regression model, the expression data of these candidate miRNAs in the validation phase were combined to construct diagnostic panels. We found that a combination of the four miRNAs (miR-18a-5p, miR-181b-5p, miR-138-5p, and miR-141-3p) emerged the best panel to screening RCC and the panel was following the formula: Logit(P) = 5.523 + 4.682 × miR-18a-5p + 2.529 × miR-181b-5p-8.527 × miR-138-5p - 5.976 × miR-141-3p. The AUC of the four-miRNA panel was 0.908 (95% CI: 0.852–0.948; sensitivity = 80.77%, specificity = 88.89%; Figure 4).

### Bioinformatics Analysis of the Candidate miRNAs

**FIGURE 5:**
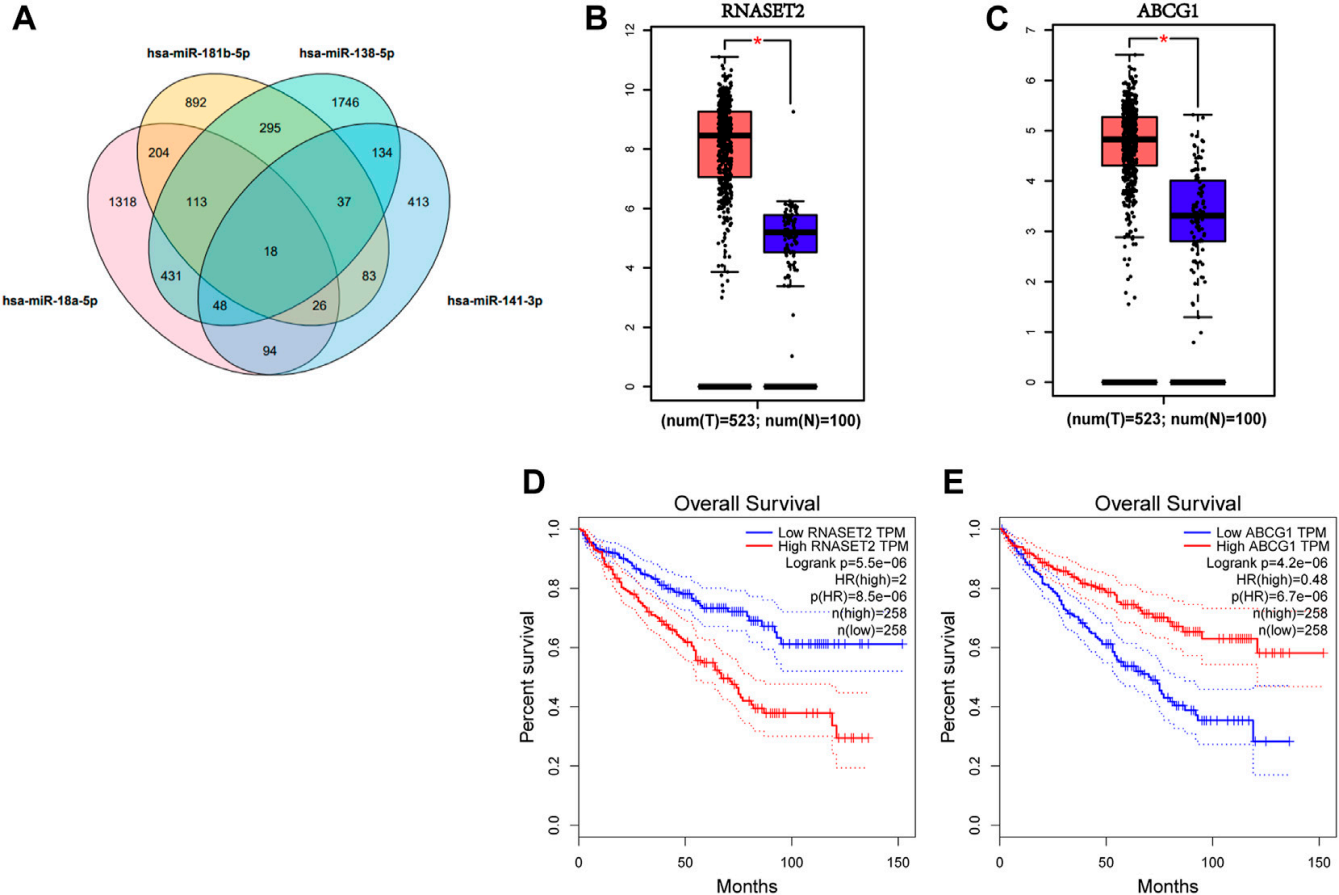
Target gene prediction of the four candidate miRNAs by miRWalk 3.0. **(A)**. 1,435 genes predicted in more than two miRNAs were considered potential targets. Expression levels of these 18 genes predicted by four candidate miRNAs in 523 RCC patients and 100 NCs were analyzed by GEPIA. **(B)** RNASET2 and **(C)** ABCG1 were dysregulated with |log2FC| > 1.5, *p* < 0.01. **(D)** RNASET2 and **(E)** ABCG1 were associated with the prognosis of RCC. T means tumor; N means normal control.

A total of 1,435 genes targeted by miR-18a-5p, miR-138-5p miR-141-3p, and miR-181b-5p were predicted by miRWalk3.0, if the prediction of these genes was targeted by more than two miRNAs, as shown in Figure 5A. Also, the network of miRNA-genes is shown in Supplementary Figure S2. The expression of the 18 genes predicted in all candidate miRNAs in RCC patients was determined by data from TCGA and the GTEx projects through GEPIA databases (Tang et al., 2017). We found that RNASET2 and ABCG1, significantly differentially expressed in RCC patients compared to NCs under the standard with *p* < 0.01 and |log2FC| Cutoff > 1.5, were considered potential target genes of the four-miRNA panel (Figures 5B,C). As shown in Figures 5D,E, RNASET2 and ABCG1 were significantly associated with the prognosis of RCC by the analysis of GEPIA databases. We also conducted a prognosis analysis of other genes predicted in all candidate miRNAs. S100A7A and NKAIN3 could not analyze because the sample size is insufficient at setting strict thresholds. As shown in Supplementary Figure S3, COX18, DCTN5, HEMK1, IL17RD, POGK, SMAD2, SRP19, TMEM154, and ZNF562 were also associated with the prognosis of RCC.

**FIGURE 6:**
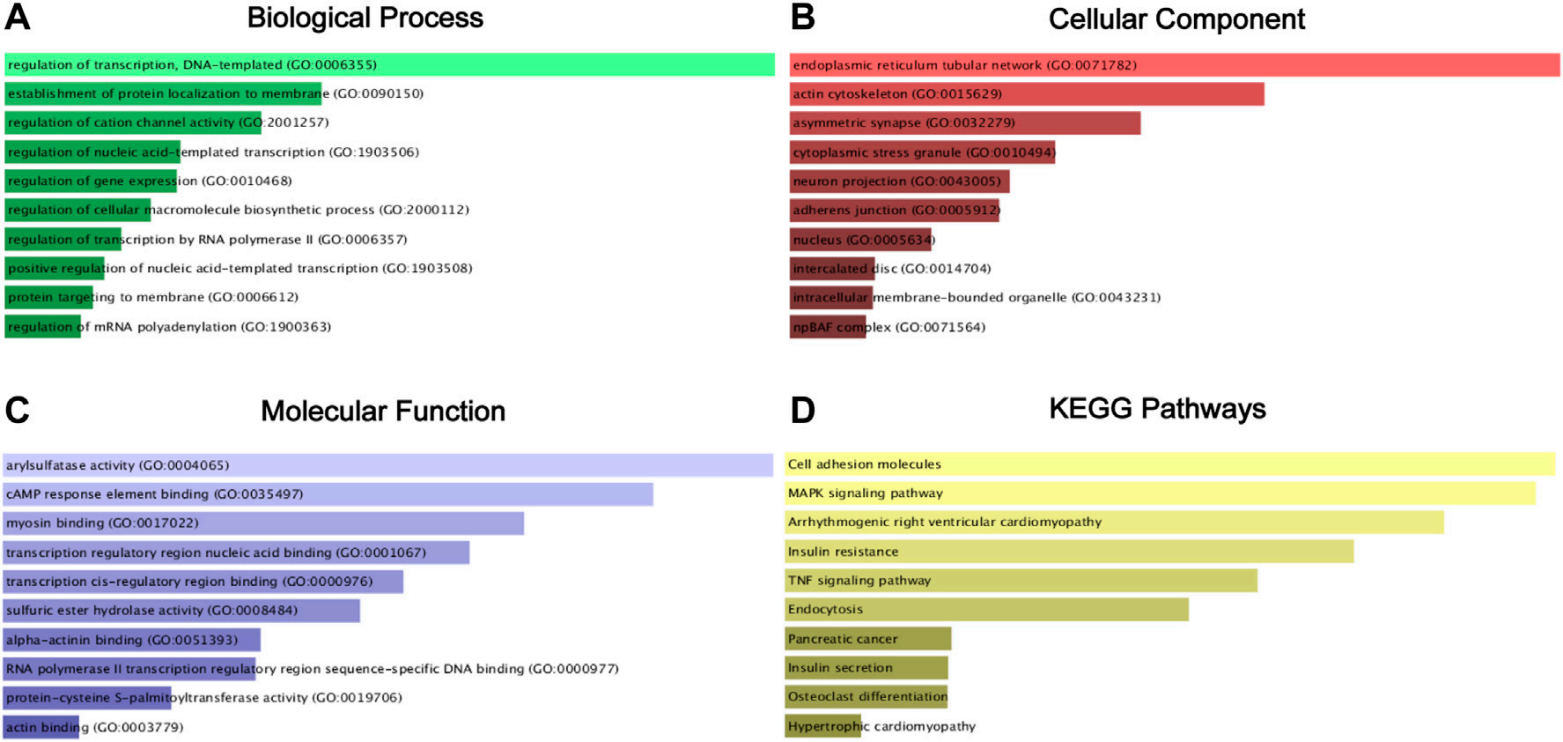
GO functional annotation and KEGG pathway enrichment analysis of the target genes of miR-18a-5p, miR-138-5p, miR-141-3p, and miR-181b-5p. **(A)** Biological process (BP) analysis; **(B)** cellular component (CC) analysis; **(C)** molecular function (MF) analysis; **(D)**. KEGG pathway enrichment analysis.

Then, the 1,435 targeted genes were put into the Enrichr database for GO annotation and KEGG pathway enrichment analysis. The top 10 enriched GO terms in each GO functional annotation including biological process (BP), cellular component (CC), and molecular function (MF) are shown in Figures 6A–C, including regulation of transcription, DNA-templated (GO:0006355), the establishment of protein localization to the membrane (GO:0090150), and regulation of cation channel activity (GO:2001257) in the biological process category; endoplasmic reticulum tubular network (GO:0071782), actin cytoskeleton (GO:0015629), and asymmetric synapse (GO:0032279) in the cellular component category; arylsulfatase activity (GO:0004065), cAMP response element-binding (GO:0035497), and myosin binding (GO:0017022) in the molecular function category. As shown in Figure 6D, enrichment pathways in KEGG pathway analysis included cell adhesion molecules, MAPK signaling pathway, and TNF signaling pathway.

### Survival Analysis of the Candidate miRNAs

**FIGURE 7:**
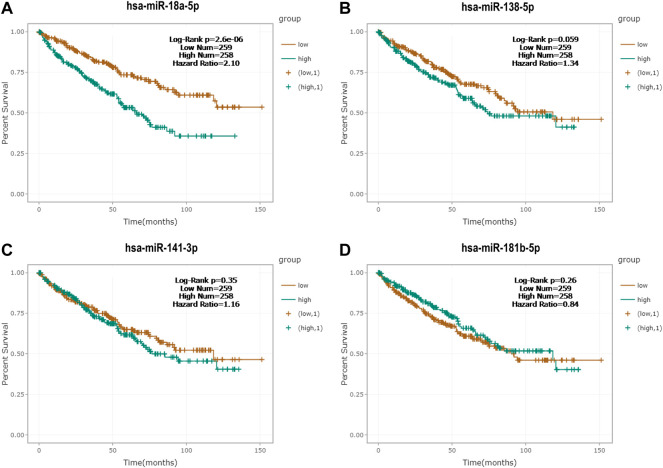
Kaplan‐Meier survival curves of **(A)** miR-18a-5p, **(B)** miR-138-5p, **(C)** miR-141-3p, and **(D)** miR-181b-5p by the ENCORI database.

Kaplan–Meier survival analysis and log-rank test were generated by the survival data of RCC patients from the ENCORI database. As shown in Figure 7 by the Kaplan–Meier survival curves, miR-18a-5p was significantly associated with the survival rate of RCC, and the higher expression level of miR-18a-5p was associated with the worse prognosis of RCC patients.

## Discussion

In recent years, RCC has been a major health problem and is one of the most malignant tumors around the world. Also, the survival rate of RCC depends on the clinical stage at diagnosis (Padala et al., 2020). Thus, early detection could improve the overall survival rate of RCC patients significantly. The miRNA screening based on serum is a widely applicable and novel diagnostic tool, especially with its non-invasive nature. In our study, the three-phase study was designed to identify miRNAs in serum that might have detection capabilities on RCC. First, we selected candidate miRNAs from pieces of literature related to RCC and screened the relative expression level by the ENCORI database. Also, 12 miRNAs were chosen as candidate miRNAs for the next investigation. After confirmation of these candidate miRNAs in the training and the validation phase, six miRNAs (miR-18a-5p, miR-138-5p, miR-141-3p, miR-181b-5p, miR-200a-3p, and miR-363-3p) in serum were significantly dysregulated between RCC patients and NCs. Moreover, we identified a four-miRNA panel, namely, miR-18a-5p, miR-138-5p miR-141-3p, and miR-181b-5p, which might be performed as a potential serum biomarker for screening RCC, with AUC = 0.908; 95% CI: 0.852–0.948; sensitivity = 80.77%, specificity = 88.89%; (Figure 5).

In the non-invasive and novel panel for screening RCC, our study showed that miR-18a-5p was significantly associated with the survival rate of RCC by Kaplan–Meier survival analysis. There was evidence showing that miR-18a-5p was elevated in RCC cell lines and tissues, produced a marked effect as an oncogene, and functioned as a prognostic biomarker in RCC (Zhou et al., 2018). Also, through the Cox regression model, a 3-miRNA signature including miR-130b, miR-223, and miR-18a performed as a potential prognostic biomarker for patient survival with clear cell renal cell carcinoma (Luo et al., 2019). Sunitinib is one kind of targeted therapy with tyrosine kinase inhibitors (TKIs), which is indicated as first-line therapy for metastatic RCC and suppressed tumor cell proliferation and angiogenesis. The miR-18a-5p was significantly decreased in sunitinib-resistant RCC cell lines and might contribute to sunitinib resistance in RCC cells (Yamaguchi et al., 2017).

The miR-138-5p had the largest diagnostic ability among the four candidate miRNAs, and it was a potential serum biomarker for screening RCC (AUC = 0.771; 95% CI: 0.698 to 0.834; Figure 4D). Yang Liu et al. indicated that miR-138-5p involving the transcription of transcription regulator family member A (SIN3A) and the following regulation of the Notch signaling pathway inhibited proliferation and invasion of renal clear cell carcinoma (Liu and Qu, 2021). Also, miR-138-5p functioned as a tumor-suppressive factor by targeting transmembrane protein 40 (TMEM40) directly in renal clear cell carcinoma (Liu et al., 2020). Curcumin played an important role in the progress of RCC, and curcumin suppressed tumorigenesis of RCC *via* regulating the circ-FNDC3B/miR-138-5p/IGF2 axis *in vitro* and *in vivo* (Xue et al., 2021). Also, miR-138-5p was sponged by circ-ZNF609 to regulate FOXP4 expression in RCC, and the circ-ZNF609/miR-138-5p/FOXP4 regulatory network played a role in the pathogenesis of RCC (Xiong et al., 2019). Thus, miR-138-5p has a vital role in the development of RCC.

Pieces of evidence showed that miR-141-3p might play the role of a tumor-suppressive gene in RCC. For instance, Yifei Liu et al. showed that miR-141-3p was remarkably downregulated and could suppress migration, invasion, proliferation, and apoptosis of renal clear cell carcinoma by regulating NIMA (never in mitosis, gene A)-related kinase-6 (NEK6) (Liu et al., 2022). Also, the overexpression of miR-141-3p inhibited the migration, invasion proliferation, clonogenicity, apoptosis, and tumor development in a xenograft mouse model of RCC (Dasgupta et al., 2020). The miR-141-3p had tumor-suppressive effects by reducing the migration and invasion of RCC cells, and co-overexpression of the miR-145-5p and miR-141-3p could result in increased inhibition of cell migration (Liep et al., 2016). Also, miR-141-3p was involved in nephrogenesis, and miR-141-3p might be related to posttranscriptional repression of the RNA decapping enzyme Dcp2 expression during renal development (Zhang et al., 2018).

As for miR-181b-5p, more and more studies have focused on the role of miR-181b-5p in many types of cancer. For example, the overexpression of miR-181b-5p enhanced cholangiocarcinoma cell invasion, migration, and proliferation by regulating PARK2 *via* the PTEN/PI3K/AKT signaling pathway (Jiang et al., 2022). Also miR-181b-5p regulated anticancer drug resistance and autophagy in gastric cancer cells (Yeon et al., 2021). Also, miR-181b-5p encapsulated in tumor-derived extracellular vesicles could affect angiogenesis to promote the tumorigenesis and metastasis of esophageal squamous cell carcinoma (Wang et al., 2020). In the study of RCC, TGF-β1 affected the expression of miR-181b-5p to regulate the proliferation and progression of RCC (Hanusek et al., 2022). Also, miR-181b-5p was upregulated by circular RNA circCSNK1G3 to promote tumor growth and metastasis in RCC *via* the TIMP3-mediated epithelial-to-mesenchymal transition (EMT) process (Li et al., 2021).

Our study showed that ABCG1 and RNASET2 were considered the potential target genes of the four-miRNA panel and were significantly associated with the prognosis of RCC. Fucheng Meng et al. researched prognostic and diagnostic value of ABCG1 and showed that ABCG1 was a potential prognostic and diagnostic biomarker in renal clear cell carcinoma (Meng et al., 2021). RNASET2 was focused on many cancers but few in RCC. The ubiquitination and degradation of FBXO6-mediated RNASET2 regulated the progress of ovarian cancer (Ji et al., 2021). RNASET2 was downregulated in gastric adenocarcinoma, and the expression of RNASET2 could be performed as a biomarker for detecting the early stage of gastric adenocarcinoma (Zeng et al., 2020). The role of ABCG1 and RNASET2 in RCC needs to be confirmed by more studies.

Our study conducted GO annotation and KEGG pathway enrichment analysis for target genes. In the biological process terms, the regulation of transcription, DNA-templated, nucleic acid-templated transcription, gene expression, and cellular macromolecule biosynthetic process were common biological processes in the development of cancer and RCC (Cree, 2011; Maher, 2013; Hsieh et al., 2017; Huang et al., 2022). As for the regulation of transcription by RNA polymerase II, previous studies showed that von Hippel–Lindau regulated hydroxylation and ubiquitylation of RNA polymerase II in RCC cells, and the hydroxylation of RNA polymerase II-dependent on von Hippel-Lindau might play a carcinogenesis role in RCC (Haffey et al., 2009; Yi et al., 2010). Alternative polyadenylation of mRNA could impact cancer development, regulate the tumor immune microenvironment in RCC, and affect RCC survival outcomes (Yalamanchili et al., 2020; Zhong et al., 2022). Transcription regulatory region nucleic acid binding and transcription cis-regulatory region binding in the molecular function catalog were common in the development of cancer (Cree, 2011). CAMP response element-binding protein (CREB) played an important role in RCC. CREB1 was an oncogenic transcription factor and affected the tumorigenesis of RCC by regulating some miRNAs (Li et al., 2016; Friedrich et al., 2020a; Friedrich et al., 2020b). In the cellular component terms, the endoplasmic reticulum tubular network, adherens junction, nucleus, and intracellular membrane-bounded organelle were related to the cancer cells (Cree, 2011; Wang et al., 2019). The npBAF complex influenced human phenotypic variation, and the BAF complex could cause tumor growth by regulating the transcriptome of the stem (Staahl and Crabtree, 2013; Alfert et al., 2019).

Enrichment pathways in the KEGG pathway analysis of targeted genes included cell adhesion molecules, MAPK signaling pathway, and TNF signaling pathway. Also, current research showed that they played a huge role in the progression of RCC. It was Anbang Wang et al. who revealed that cell adhesion–related molecules were related to the progression and poor prognosis of RCC, such as EGFR, CD44, FN1, and CD86 (Wang et al., 2019). Low-vitamin D status is related to an increased risk of RCC, and Shen Xu et al. showed a mechanistic explanation for the association between vitamin D and adhesion molecules in patients with RCC (Xu et al., 2019). Also, the study showed that complement component 1q subcomponent binding protein (C1QBP) could suppress RCC metastasis by regulating cell adhesion molecules (Wang et al., 2017). The inhibition of MAPK signaling pathways might inhibit the growth of RCC by destroying the tumor vascular system (Huang et al., 2008). Also, tribbles pseudokinase 3 (TRIB3) regulated tumor progression by activating the MAPK signaling pathway to accelerate the proliferation, migration, and invasion of RCC (Hong et al., 2019). Also, pleckstrin homology domain-containing O1 (PLEKHO1) might promote the development of RCC by the Hippo and MAPK/JNK pathways *in vitro* and *in vivo* (Yu et al., 2019). Also, miR-106 b could promote RCC progression by targeting Capicua through MAPK signaling (Miao et al., 2019). And mTOR pathway inhibitors induced MAPK escape cell death and cells became resistant to mTOR inhibitors, and then the combination of mTOR and MAPK inhibitors would get better treatment and outcome for RCC patients (Chauhan et al., 2016). Sheng-Tang Wu et al. showed that the TNF-α signaling pathway was associated with the tumorigenesis of RCC, and TNF-α induced EMT of RCC cells through a nuclear factor-kappa B-independent mechanism (Wu et al., 2011).

Although our results are meaningful, there are still some limitations to be solved. First, the number of participants enrolled in our study was relatively small, and the effect would be better if the external validation set was added to the study. Second, our study only chose 12 initial miRNAs, but there were many dysregulated miRNAs in serum and related to RCC. Furthermore, studies were necessary to study the value of other miRNAs in the screening of RCC and explore their value as clinical biomarkers. Also, we could explore the role of RNASET2 and ABCG1 in the development of RCC and the association with candidate miRNA further.

In summary, we found six miRNAs in serum that were dysregulated significantly between renal cell carcinoma patients and normal controls. We also assessed their ability to screen RCC, and the 4-miRNA panel might be considered a non-invasive and novel biomarker for screening RCC (AUC = 0.908). Target genes ABCG1 and RNASET2 may be potential biomarker therapeutic targets in RCC. Also, the MAPK signaling pathway might play an important role in the process of RCC development.

## Data Availability

The datasets presented in this study can be found in online repositories. The names of the repository/repositories and accession number(s) can be found in the article/Supplementary Material.
