# Supplementary material for: A Four-MicroRNA Panel in Serum as a Potential Biomarker for Screening Renal Cell Carcinoma
Source: Front Genet. 2022 Jul 22;13:897827. doi: 10.3389/fgene.2022.897827 (PMC9355293; doi:10.3389/fgene.2022.897827)
Supplement: Supplementary file 1 [file DataSheet1.docx]

Supplementary Material

# Supplementary Table

| miRNA | Cancer number | Normal number | Fold Change | P-Value | FDR |
| --- | --- | --- | --- | --- | --- |
| hsa-miR-18a-5p | 517 | 71 | 2.29 | 7.7e-15 | 1.20e-13 |
| hsa-miR-93-5p | 517 | 71 | 2.49 | 3.5e-42 | 2.50e-40 |
| hsa-miR-138-5p | 517 | 71 | 0.23 | 3.60e-27 | 1.2e-25 |
| hsa-miR-141-3p | 517 | 71 | 0.15 | 5.2e-74 | 1.7e-71 |
| hsa-miR-181b-5p | 517 | 71 | 2.79 | 3.6e-37 | 1.9e-35 |
| hsa-miR-200a-3p | 517 | 71 | 0.46 | 6.6e-23 | 1.80e-21 |
| hsa-miR-223-3p | 517 | 71 | 2.29 | 4.6e-22 | 1.2e-20 |
| hsa-miR-363-3p | 517 | 71 | 0.33 | 2.10e-25 | 6.4e-24 |
| hsa-miR-429 | 517 | 71 | 0.29 | 2.20e-32 | 9.20e-31 |
| hsa-miR-452-5p | 517 | 71 | 4.56 | 4.4e-41 | 2.9e-39 |
| hsa-miR-532-3p | 517 | 71 | 0.28 | 1.9e-53 | 2.2e-51 |
| hsa-miR-590-5p | 517 | 71 | 2.11 | 1.5e-24 | 4.3e-23 |

**Supplementary Table 1.** The differential expression data of 12 candidate miRNAs analyzed by ENCORI database.

# Supplementary Figures


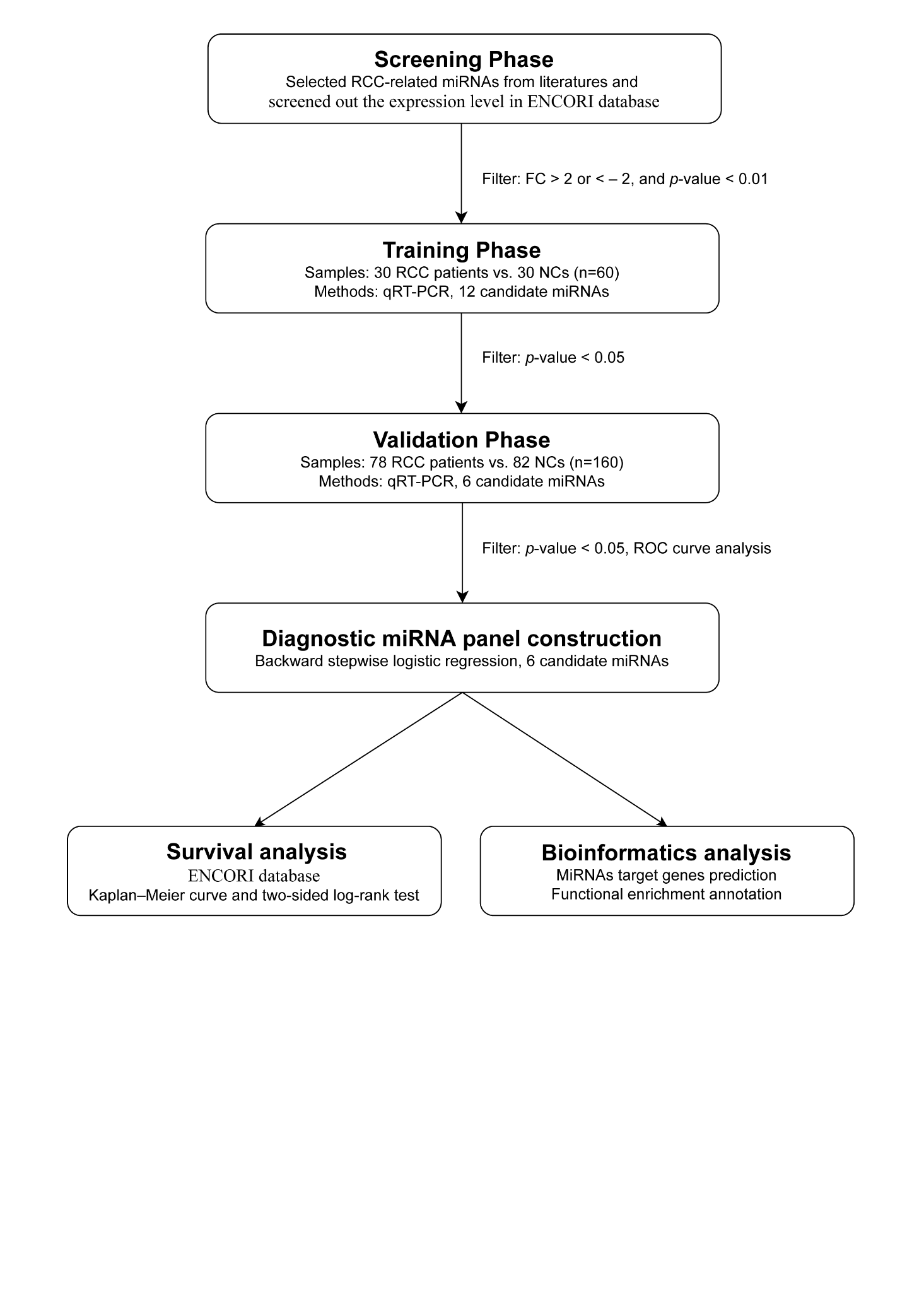
**Supplementary Figure 1.** Study's flowchart.


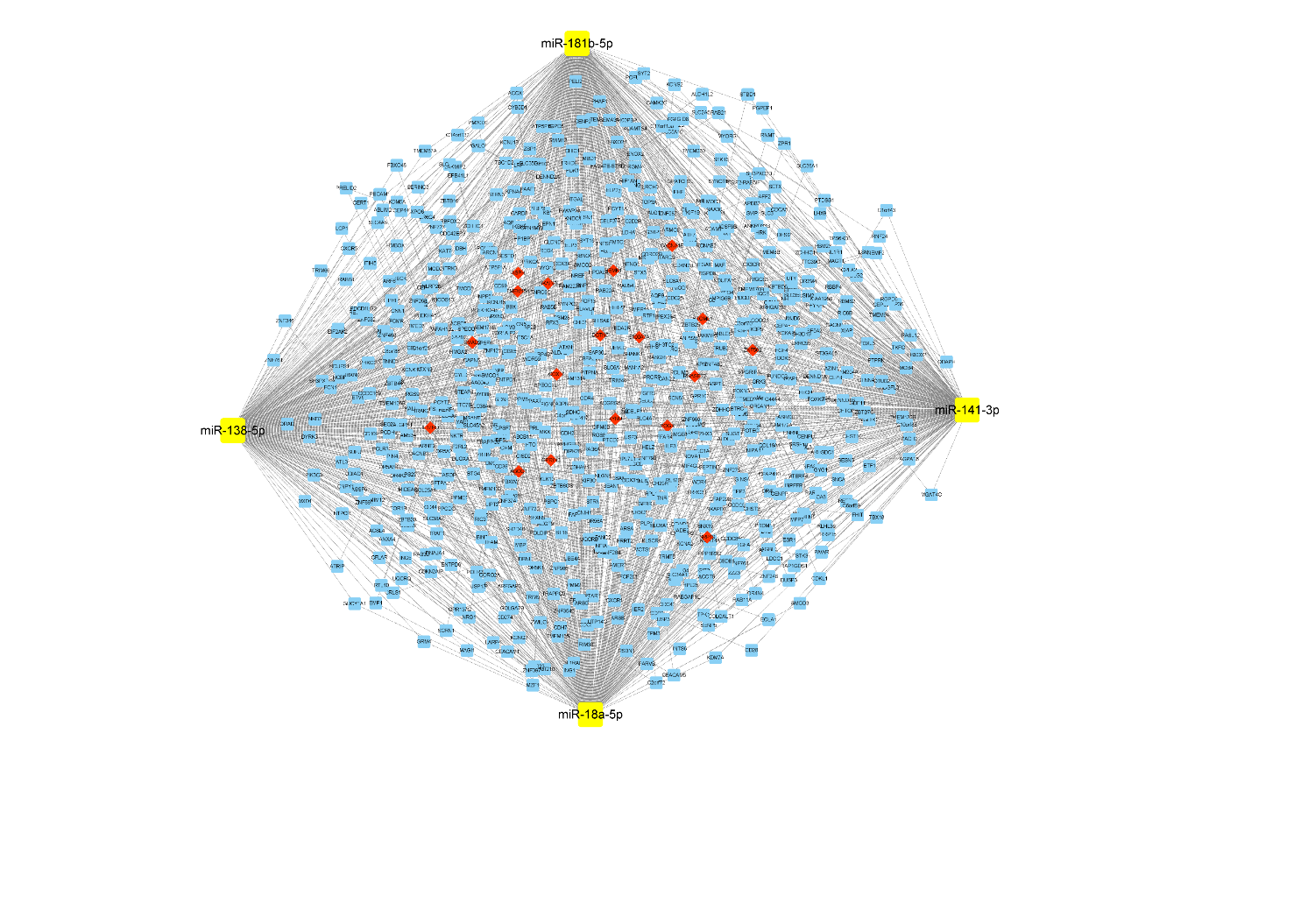


**Supplementary Figure 2.** The network of miRNAs-genes. The yellow is candidate miRNA. The red is target gene predicted in all candidate miRNAs. The blue is other target gene.


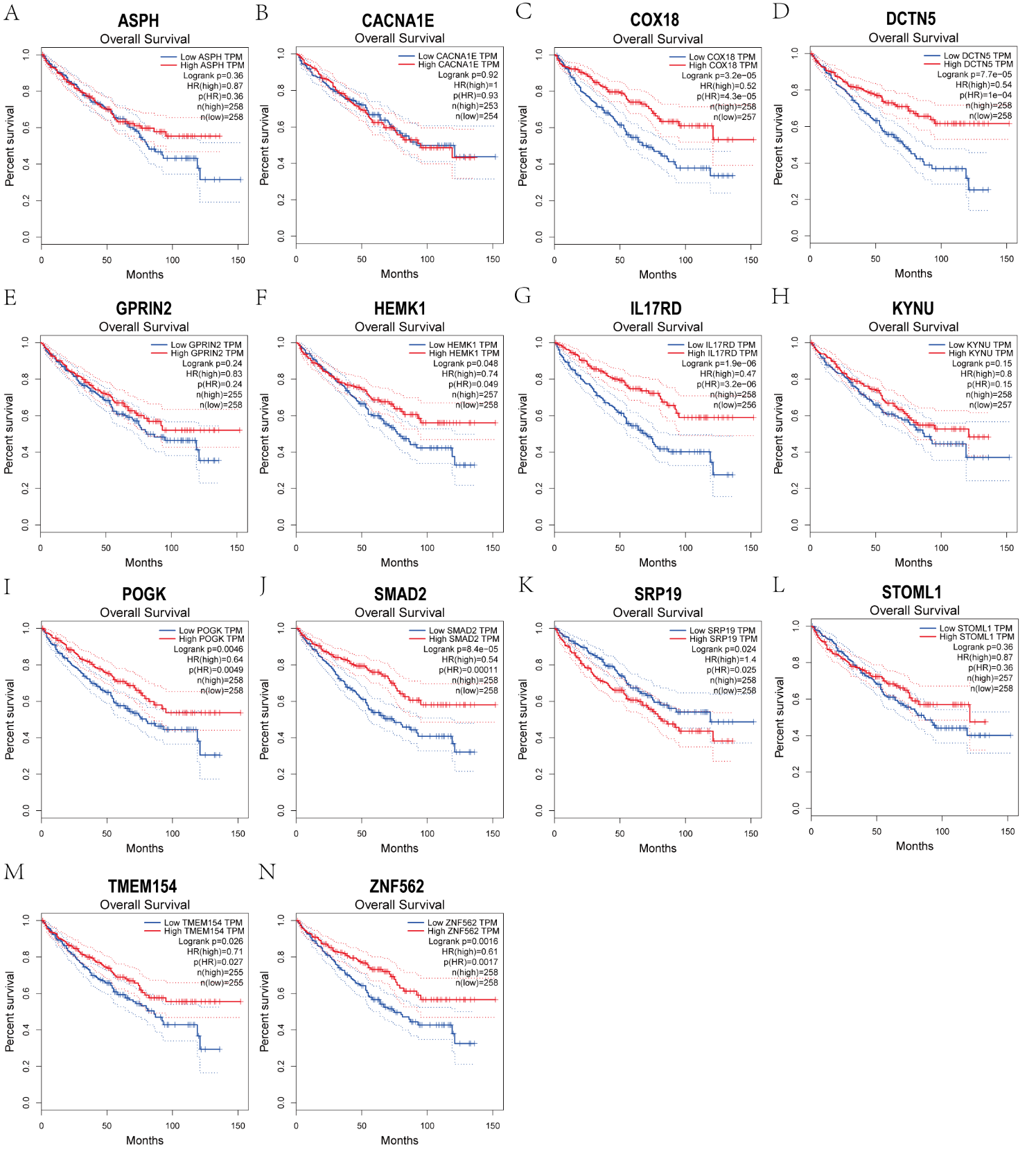


**Supplementary Figure 3.** The prognostic analysis of other 14 genes predicted in all candidate miRNAs by GEPIA. COX18(C), DCTN5(D), HEMK1(F), IL17RD(G), POGK(I), SMAD2(J), SRP19(K), TMEM154(M), and ZNF562(N) were associated with the prognosis of RCC with p<0.05. ASPH(A), CACNA1E(B), GPRIN2(E), KYNU(H), and STOML1(L) were not associated with the prognosis of RCC.
